# Novel Antivirals in Clinical Development for Chronic Hepatitis B Infection

**DOI:** 10.3390/v13061169

**Published:** 2021-06-18

**Authors:** Lung-Yi Mak, Wai-Kay Seto, Man-Fung Yuen

**Affiliations:** 1 Department of Medicine, Queen Mary Hospital, The University of Hong Kong, Pokfulam Road 102, Hong Kong, China; loeymak@gmail.com (L.-Y.M.); wkseto@hku.hk (W.-K.S.); 2 State Key Laboratory of Liver Research, The University of Hong Kong, Hong Kong, China

**Keywords:** functional cure, CpAMs, gene silencing, antiviral therapy, immunomodulation, STOPS, therapeutic vaccines, checkpoint inhibitors, ImmTAV

## Abstract

Globally, chronic hepatitis B (CHB) infection is one of the leading causes of liver failure, decompensated cirrhosis, and hepatocellular carcinoma. Existing antiviral therapy can suppress viral replication but not fully eradicate the virus nor the risk of liver-related complications. Novel treatments targeting alternative steps of the viral cycle or to intensify/restore the host’s immunity are being developed. We discuss novel drugs that have already entered clinical phases of development. Agents that interfere with specific steps of HBV replication include RNA interference, core protein allosteric modulation, and inhibition of viral entry or viral protein excretion (NAPs and STOPS). Agents that target the host’s immunity include toll-like receptor agonists, therapeutic vaccines, immune checkpoint modulators, soluble T-cell receptors, and monoclonal antibodies. Most have demonstrated favorable results in suppression of viral proteins and genomic materials (i.e., HBV DNA and/or pre-genomic RNA), and/or evidence on host-immunity restoration including cytokine responses and T-cell activation. Given the abundant clinical experience and real-world safety data with the currently existing therapy, any novel agent for CHB should be accompanied by convincing safety data. Combination therapy of nucleos(t)ide analogue, a novel virus-directing agent, and/or an immunomodulatory agent will be the likely approach to optimize the chance of a functional cure in CHB.

## 1. Introduction

Chronic hepatitis B infection (CHB) affects 292 million people globally [1], and is a major risk factor of liver-related diseases including liver failure, cirrhosis, and hepatocellular carcinoma (HCC); and mortality. Many patients with CHB require antiviral therapy to suppress viral replication in order to reduce the risk of liver-related complications. Nucleos(t)ide analogues (NAs) and pegylated interferon (PEG-IFN) are the currently available treatment options in CHB. These agents, especially NAs, are highly effective in achieving normalization of alanine aminotransferase (ALT), hepatitis B virus (HBV) DNA suppression [2,3], and risk reduction in HCC and mortality [4,5,6]. Due to the high rate of virological relapse post-NA cessation in patients with HBeAg-negative CHB [7,8], most NA-treated patients require lifelong therapy, which creates issues of cost, adherence, and risks of therapy-related adverse events [9]. Moreover, the risk of liver-related complications is not entirely eliminated despite long-term therapy. In view of these concerns, the goals of therapy have been redefined and new treatment approaches are being developed, which will be reviewed in the following sections.

## 2. Functional Cure: The Preferred Treatment Endpoint

Even with wide availability of NAs that are potent and safe, there is a pressing need for additional treatment due to the fact that the existing therapy cannot eliminate the residual risks of liver-related complications and the indefinite duration of long-term antivirals. The goal of developing novel therapies is to promote a cure in patients with CHB, which refers to elimination of the virus from the host, following which antiviral therapy can be stopped; i.e., finite therapy, with minimal risks of virological relapse and ongoing liver damage, which eventually will reduce the risk of cirrhosis and HCC. Table 1 shows the preferred therapeutic endpoints in CHB. *Partial cure* refers to sustained virological suppression after stopping NA, which is a better treatment endpoint compared to virological suppression with long-term NA, as patients can remain drug-free if it is achieved. *Functional cure* is a superior treatment endpoint to partial cure, which refers to sustained seroclearance of hepatitis B surface antigen (HBsAg) with or without seroconversion of antibody to HBsAg (anti-HBs). NA has been shown to reduce intrahepatic covalently closed circular DNA (cccDNA) from 7.28 to 0.57 copies/cell upon 1 year of treatment, and even below detection limit upon ≥6 years of NA treatment, although expression of HBsAg was not completely suppressed [10]. It is believed that with time and appropriate therapeutic strategy, elimination of the cccDNA pool, i.e., *complete cure*, will become feasible. Due to the existence of integrated HBV DNA, a *sterilizing cure* is clearly impossible at present and is not expected to be achievable within a short period of time. Therefore, functional cure is deemed an achievable goal for the time being, among the few therapeutic endpoints, and should this be the primary endpoint; while partial cure is regarded as an intermediate goal for phase 3 trials in CHB [11].

Achieving a functional cure is associated with better clinical outcomes. Firstly, antiviral treatment can be safely stopped (except in patients with cirrhosis with detectable DNA) with a low risk of HBsAg seroreversion or virological rebound [12,13]. Secondly, patients who achieve a functional cure, especially if onset of HBsAg seroclearance is below age of 50 years old, are at a significantly lower risk of HCC [14,15,16]. Thirdly, liver fibrosis will regress and the risks of liver decompensation, of need for liver transplantation, and of death are significantly lowered in patients who have achieved functional cure [17,18,19]. In view of the clear evidence of benefits brought by functional cures, and the fact that achieving HBsAg seroclearance is a rare event with the current therapy (around 1–2% per year), novel therapies are aimed at improving the chance of achieving a functional cure, or at least achieving a partial cure in patients expected to be on NA for an indefinite duration.

## 3. Novel Therapeutic Approaches

**Figure 1:**
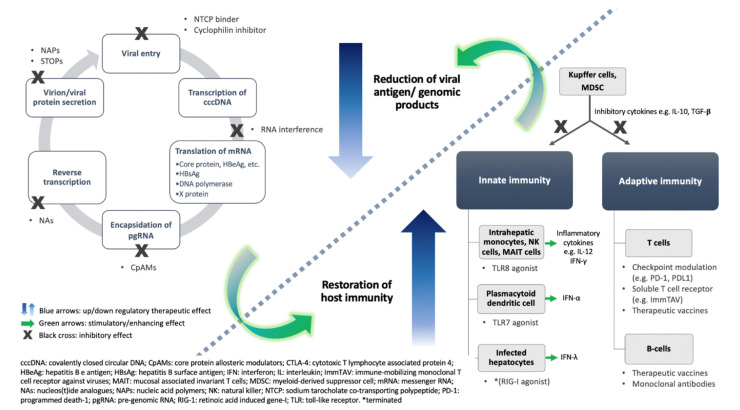
Schematic diagram illustrating the therapeutic approaches currently in clinical development in chronic hepatitis B infection. Left panel: virus-directed agents target different steps of the viral replicatory cycle, including viral entry, RNA interference, capsid formation and pgRNA encapsidation, DNA synthesis, and viral protein secretion. Although NAs are already in clinical use, they will remain the backbone of combination therapy in the context of novel antiviral treatment. Right panel: immune modulatory agents restore the host immunity at both innate and adaptive systems, which are interlinked by inflammatory cytokines and specific cell types. *RIG-I agonists once entered a phase 2 clinical trial but this was terminated due to a safety issue. The two approaches are intercalated as represented by the curved green arrows and the dotted line.

Novel therapeutic approaches aim to achieve sustained viral suppression and restoration of anti-HBV immunity through a finite duration of therapy [20,21]. They can be divided into two types: inhibition of alternative steps of viral replication (i.e., virus-directing agents) and direct enhancement of host immunity (immunomodulatory agents). The two approaches are closely intercalated, as HBV itself plays a major role in the immunopathogenesis of CHB. In neonatal infection, the HBV antigens behave as neo-self-antigens and elicit tolerance as opposed to activation of HBV-specific T cells, which will lead to clonal deletion or clonal downregulation of high-affinity T-cell clones that are specific to all structural and non-structural HBV antigens (HBs, HBe/HBc, Pol, and X) [22]. Without effective antiviral functions, the virus persists in the body, and chronic exposure to the viral proteins results in impaired activation of toll-like receptor pathways [23] and lymphocyte dysfunction [24] through mechanisms including clonal anergy, clonal ignorance, T-regulatory activity, and immune checkpoints [25]. In contrast to patients with self-limiting acute HBV infection, CHB patients do not have strong, multiple, and sustained cytotoxic T lymphocyte responses [26,27]. Enhanced and sustained expressions of different types of inhibitory markers on CD8^+^T cells have been reported, which include programmed cell-death protein-1 (PD-1), T-cell immunoglobulin and mucin-domain-containing 3 (TIM-3), cytotoxic T lymphocyte-associated antigen-4 (CTLA-4), and CD244 [28,29]. In CHB, B lymphocytes, albeit with static numbers in the peripheral blood, are functionally deficient and exhibit atypical phenotypes (CD21^-^ CD27^-^) with enhanced PD-1 expression, resulting in impaired production of neutralizing antibodies [30]. HBsAg also enhances regulatory T-cell [Treg (CD4^+^CD25^+^FoxP3^+^)] and myeloid-derived suppressor cells (MDSC) activity [31,32]. These mechanisms (i.e., chronic viral antigen exposure and the resultant numerical and/or functional depletion of cells in both innate and adaptive immune systems) have been proposed to contribute to the HBV chronicity, i.e., persistence of HBV beyond 6 months without development of polyclonal HBV-specific lymphocytes [2]. It is generally accepted that HBsAg seroclearance is the result of regaining immunologic control of HBV from the immune exhaustion exerted by the huge numbers of viral proteins. Therefore, with the help of virus-directing agents to reduce the viral proteins, the host immunity can be restored and, thus, in turn the virus can be controlled. On the other hand, immunomodulatory agents directly suppress viral replication via enhancement of cellular effector functions (Figure 1). In the following sections, only agents that are already in the clinical phases of development will be discussed. As mentioned above, a functional cure is the desirable endpoint and should be achieved in ≥30% of patients for drugs tested in phase 3 trials [11]. For early-phase development, i.e., phase 1 and 2 trials, no recommendations or guidance have been proposed but are usually substituted by achieving a pre-defined HBsAg level or magnitude of HBsAg decline. This is likely to be related to the fact that HBsAg seroclearance is preceded by a low HBsAg level [33,34], and only agents that demonstrate potent effect in suppression of HBsAg in early-phase trials will show potential to achieve the desirable endpoint for phase 3 trials. Therefore, HBsAg decline is the most frequently assessed parameter for assessing the efficacy of anti-HBV drugs in the trials discussed below. In addition to exploring whether novel agents are able to achieve a higher rate of functional cure, it would be highly appreciable if novel agents could also have a more long-term effect on cccDNA reduction.

### 3.1. Virus-Directing Agents

The replication of HBV involves many different steps, including viral entry, transcription, translation, encapsidation, DNA synthesis, and virion export. These are the target sites for anti-HBV drugs to act on — competing for the sodium taurocholate co-transporting polypeptide (NTCP), interference of RNA transcription, inhibition of functional capsid formation/encapsidation, DNA synthesis, and viral protein export. Table 2 summarizes the virus-directing agents for CHB that are currently in clinical phases of development.

#### 3.1.1. Inhibition of Viral Entry

Sodium taurocholate co-transporting polypeptide (NTCP) is a hepatic bile acid cotransporter and is the receptor for HBV entry, which uses its surface lipopeptide pre-S1 for docking to the hepatocyte via NTCP [35]. Myrcludex B, now named Bulevertide, is a lipomyristolated peptide containing 47 amino acids of the pre-S1 domain of the HBV large surface protein that competes with natural HBsAg for NTCP binding and thereby inhibits viral entry. This drug has been approved for medical use in the European Union in July 2020 for patients with chronic hepatitis B/D coinfection. HBV DNA was significantly reduced compared to PEG-IFN monotherapy after 24 weeks of combination therapy with Myrcludex B with PEG-IFN in patients with hepatitis B/D coinfection. Although HBsAg reduction was not significantly different between the two treatment arms initially, continuation of combination therapy until 48 weeks was associated with a higher proportion of patients achieving HBsAg reduction >1 log IU/mL and/or HBsAg negativity compared to PEG-IFN monotherapy [36]. Myrcludex B needs to be administered subcutaneously, and is in general well-tolerated. Higher doses of Myrcludex B (IC_50_ 47 nM) would affect bile acid transportation and there was once a concern that it would lead to increased risks of adverse reactions related to bile acid accumulation. Fortunately, effective viral entry inhibition can be achieved at much lower doses (IC_50_ 80 pM, i.e., 500 folds lower than the dose that would affect the bile acid metabolism) [37], thus theoretically related adverse events, e.g., pruritus or diarrhea, were not observed at the therapeutic doses used for CHB patients in the clinical trials [38]. CRV431 is a cyclophilin inhibitor that inhibits HBV entry without interfering with the NTCP transporter [39]. When given orally in transgenic mice, CRV431 led to a significant reduction in liver HBV DNA levels and serum HBsAg level without toxicities [40]. The exact anti-HBV mechanisms remain unknown but are believed to be the neutralizing effect on the peptidyl–prolyl isomerase activity resulting in interference with protein unfolding. CRV431 is currently undergoing phase 1 study.

#### 3.1.2. Interference of RNA

RNA interference (RNAi) is a naturally occurring and biologically conserved mechanism for specific post-transcriptional gene silencing [41,42]. RNAi can directly target HBV transcripts and induce degradation, thereby leading to gene silencing. Small interfering RNAs (siRNAs) are structurally duplex (ds) RNA strands with guide and passenger RNA strands of 23 nucleotide and 21 nucleotides, respectively. After association with the Argonaute (AGO)-RNA-induced silencing complex, the passenger strand is removed and the guide strand leads the AGO to the target complementary mRNA and induces mRNA cleavage [43]. As siRNAs cannot enter cells by themselves, early siRNAs developed were conjugated with N-acetylgalactosamine (GalNAc), which preferentially binds to asialoglycoprotein, a receptor enriched in hepatocytes, in order to enhance hepatic uptake of the target molecule as well as to decrease off-target engagement. Antisense oligonucleotides (ASOs) are another class of gene silencers, which are structurally single DNA strands of 8 to 10 nucleotides modified to resist nucleases, which induces cleavage of the target HBV RNAs inside the nucleus and cytoplasm via RNase H1. ASOs by themselves can be taken up by all cells including hepatocytes, while the GaiNAc-conjugation improves exposure by about 10-folds [44]. Locked nucleic acid (LNA) is a type of chemical modification of the ribose sugar, which is another strategy to enhance delivery of the oligonucleotide to target binding [45,46]. GaINAc-conjugated LNA-single-stranded oligonucleotide resulted in a rapid and long-lasting reduction of HBsAg in the AAV–HBV mouse model [47]. The host RNA polymerase II uses cccDNA as a template, which contains four open reading frames (ORF) for transcribing viral RNAs that encode precore/core, polymerase, surface, and X protein using a common 3’ end. This characteristic is advantageous for RNAi therapy, because all the four viral transcripts from cccDNA and integrated DNA can be degraded using a single-target RNAi. Many RNAi therapies for CHB are currently evaluated in phase 2 clinical trials.

Most siRNAs are given at monthly doses and achieve potent and robust on-treatment HBsAg responses. JNJ-3989 [48], VIR-2218 [49], RG-6346 [50], and AB-729 [51] each led to >1 log HBsAg reduction in 85–92% subjects, and the target of nadir HBsAg < 100 IU/mL was achieved in >50% subjects. Moreover, durable post-treatment HBsAg suppression was also demonstrated after finite doses of JNJ-3989 and RG-6346. In the open-label extension cohort of the phase 1b study that involved eight CHB patients who received 4–9 weekly doses of ARC-520 (a first-in-class siRNA that was later stopped development due to death in non-human primates), HBsAg loss was seen in some patients after using multiple doses [52].

ISIS505358/GSK3228836 (GSK836) is a type of non-GaiNAc-conjugated ASO. GSK836 given subcutaneously on days 1, 4, 8, 11, 15, and 22 led to significant reductions in HBsAg, with bigger magnitudes of decline in NA-treated patients (2.51 logs) compared to NA-naïve patients (1.56 logs). Moreover, 4 out of 16 patients had transient HBsAg loss during dosing, and prolonged HBsAg loss was observed in one NA-naïve patient and one NA-treated patient after dosing of GSK836 (from day 23 to day 126 and from day 36 to day 113, respectively) [53]. ALT elevations were observed after profound suppression in HBV antigen load without dose – response relationships with RNAi dosing, which may suggest immune restoration, but more studies are required to prove this hypothesis.

#### 3.1.3. Inhibition of Functional Capsid Assembly or Encapsidation

The core protein is essential for capsid formation, encapsidation, and reverse transcription of pre-genomic RNA, virion formation, and cccDNA amplification. Core protein allosteric modulators (CpAMs) target the step of capsid formation by either forming aberrant unstable capsids (class 1) or empty capsids (class 2). Apart from reduction in the formation of mature virions, these dysfunctional capsids would also impair the nuclear recycling of rcDNA-containing nucleocapsids for cccDNA pool replenishment and epigenetic regulatory mechanisms in cccDNA transcription [54].

RO7049389 (also known as RG7907), a class 1 CpAM, was given to treatment-naïve CHB patients at daily or twice-daily oral dosing for 4 weeks, which led to median serum DNA reduction of 2.66–3.2 logs and rebound back to the baseline level after the dosing period. No significant changes were observed in the HBsAg levels [55].

GLS4, a class 1 CpAM, was coupled with ritonavir and compared with ETV monotherapy in the phase 2 trial. No patients in the ETV group achieved ≥1.5 log HBsAg decline at week 24 compared to 12.5% patients in the GLS4/ritonavir group. In addition, GLS4/ritonavir was more effective than ETV in suppression of HBV RNA (3.53 vs. 0.73 logs, respectively) and HBcrAg (1.32 vs. 0.65 logs, respectively) in previously treatment-naïve patients at week 24 [56].

Most CpAMs in development can be administered orally and are used in combination with NAs (Table 2). JNJ-6379, a class 2 CpAM, was used in combination with NA in the phase 2 trial. While JNJ-6379 + NA was effective in suppression of HBV DNA and RNA, the degree of HBsAg reduction was relatively modest (0.4 logs in HBeAg-positive treatment-naïve patients) at week 24 [57]. Six out of 31 patients had self-limiting grade 2–4 ALT elevation without derangement of coagulation profile or bilirubin, which required no treatment dose change or interruption. ABI-H0731 (Vebicorvir), a class 2 CpAM, when combined with NAs, demonstrated quicker and bigger reductions in HBV DNA and HBV RNA when compared with NA alone. In the open label extension study, 26 HBeAg-positive and 43 HBeAg-negative viral-suppressed CHB patients received Vebicorvir + NA for 76 weeks (up to 148 weeks). Twenty-three and 18 subjects, respectively, met the stopping criteria, which was defined as HBV total nucleic acids <20 IU/mL and HBeAg negative or HBeAg ≤ 5 IU/mL for ≥6 months prior to week 76. Upon cessation of therapy, 100% of them had virological relapse and none achieved HBsAg seroclearance [58]. Although Vebicorvir was well-tolerated [59], the data precludes the potential development of Vebicorvir as a finite duration of therapy. Therefore, it will not enter phase 3 clinical trial based on the developing company’s decision in February 2021 to redirect the resources to more potent next-generation CpAMs.

ALG-000184 (prodrug of ALG-001075) is another class 2 CpAM that has demonstrated 10- to 80- folds stronger antiviral activity at HBV DNA suppression compared to other CpAMs currently in clinical development in cell line models [60]. ALG-001075 demonstrated broad antiviral activity against HBV genotypes A to J, and antiviral activity was retained against most known NA resistance mutations and CpAM resistance mutations F23Y, I105F, I105T, T109M, Y118F, and T128I with only 0.5- to 3.0- fold shifts, except T33N that led to 28-fold loss of activity [60]. ALG-000184 is administered orally and is currently undergoing phase 1 clinical trial.

#### 3.1.4. Inhibition of Viral Protein Export

Nucleic acid polymers (NAPs) prevent release of HBsAg subviral particles from infected hepatocytes. The mechanism of action was investigated in a cell-based assay using human HepG2.2.15 cells. Following endosomal release, NAPs destabilize subviral particle assembly and secretion (but not Dane particles) through interaction with some uncharacterized host protein. Intracellular HBsAg accumulation is prevented by proteosomal and lysosomal degradation, which is enhanced by NAPs. Dane particles and other viral markers such as HBeAg secretion are not affected because their assembly and secretion pathways are via the multivesicular body pathway but not the endoplasmic-reticulum-Golgi intermediate compartment (ERGIC) pathway for subviral particles [61,62].

REP 2139 or REP 2165 are NAPs and were investigated in combination with PEG-IFN and NA. In the phase 2 study, combination therapy with REP 2139/REP 2165 + TDF + PEG-IFN for 24 weeks was effective in causing HBsAg < 0.05 IU/mL (60% patients), with 35% patients achieving HBsAg seroclearance [63].

S-antigen Transport-inhibiting Oligonucleotide Polymers (STOPS) share structural similarities with NAPs with stronger in vitro potency. In the preclinical studies using cell line models, ALG-010133 (STOPS) was 30- to 180-folds more potent than REP 2139 (NAP) in inhibition of HBsAg release [64]. The intracellular HBsAg was reduced upon STOPS treatment, indicating that STOPS do not simply trap HBsAg inside the cells, and the cell viability was not compromised. In addition, STOPS demonstrated pan-genotypic activity (A-D) as well as 100-fold better potency in inhibition of HBsAg production from the integrated HBV genome in cell line models [64]. ALG-010133 is currently undergoing phase 1 clinical trial. For both NAPs and STOPS, the reduction in viral antigen secretion will reduce antigen presentation on hepatocytes and theoretically should restore the function of exhausted T cells and B cells [65]. However, it is believed that exhausted T cells acquired epigenetically distinct profiles from effector T cells [66], and this may not be entirely restorable by simply reducing antigen presentation. Therefore, additional therapy following NAPs/STOPS may be needed to prime HBV-specific T cells to maximize the effect on immune restoration.

#### 3.1.5. Farnesoid X Receptor Agonist

EYP001 is an orally bioavailable second-generation non-bile acid farnesoid X receptor (FXR) agonist. The FXR nuclear receptor is highly expressed in the liver and intestine. There are two FXR response elements located in the HBV Enhancer II and core promoter region, which stimulate HBV pregenomic RNA synthesis and viral replication [67]. Modulation of FXR receptor activity by ligands alters HBV replication. In vivo and in vitro studies showed that treatment with FXR agonist inhibited the FXR proviral effect on cccDNA and the HBx-dependent inhibition of pregenomic RNA and precure RNA transcription and DNA secretion [68]. In the phase 1 study, EYP001 was shown to engage FXR as evidenced by fibroblast growth factor 19, the total bile acid precursor C4, and total bile acid changes, and led to HBsAg reduction of 0.1 log after 4 weeks of 400 mg daily dosing [69]. EYP001 is currently undergoing phase 2 clinical trial.

### 3.2. Enhancement of Host Immunity

As mentioned above, chronic viral antigen exposure weakens HBV-specific immunity and leads to virus-specific T-cell anergy, leading to immune exhaustion in the host. Novel therapies aiming to restore or enhance the host immunity are being actively developed (Table 2).

Toll-like receptors (TLRs) are pattern-recognition receptors (PRRs) that upon activation would stimulate various leukocytes in both the innate and adaptive system. GS-9620 (Vesatolimod) is a TLR7 agonist. In the phase 2 double-blind placebo-controlled trial, GS-9620 induced ≥2-fold expression of the interferon-stimulated gene (ISG15) especially in female subjects. However, no significant serum interferon (IFN) alpha expression or HBsAg decline was demonstrated [70]. GS-9688 (Selgantolimod) is a TLR8 agonist and achieved modest decline in HBsAg when given in combination with NA in the phase 2 study. GS-9688 induced dose-dependent cytokine responses (IL-12p40, IL-1RA, IFNγ) and shifts in peripheral immune cell subsets [71].

HBV-specific T-cell function can be restored by autoantibodies that block the inhibitory molecules. Nivolumab is a monoclonal antibody against PD-1 that is approved for treatment of various malignancies. When given at a reduced dose in combination with GS-4774 (a therapeutic vaccine; see below), nivolumab led to a modest reduction in HBsAg level (0.16 to 0.3 log reduction) at 12 weeks, and one patient achieved HBsAg seroclearance that was preceded by ALT flare and an increase in peripheral HBsAg-specific T cells [72].

IMC-I109V is a novel immunotherapy. It is a soluble bi-specific T-cell-engaging fusion protein comprised of a soluble affinity-enhanced T-cell receptor fused to a humanized anti-CD3 single chain variable fragment. As a type of immune-mobilizing monoclonal T-cell receptor against virus (ImmTAV), IMC-I109V TCR recognizes HBsAg presented by specific human leucocyte antigen (HLA)-A*02:01 on the surface of infected hepatocytes. Upon engagement of TCR with HBsAg, the effector domain will bind to CD3 on any surrounding T cell of a different family, which is redirected to the complex, thereby engaging the global T-cell population and compensating for the defective HBV-specific CD8 cells in CHB. In vitro study has confirmed that ImmTAV-Env (ImmTAV molecules that are specific for HLA-A*02:01-restricted HBV epitopes derived from the viral envelope proteins) can redirect T cells from healthy and HBV-infected donors toward HCC cells containing integrated DNA. The induced cytokine release was suppressible by corticosteroid, and the redirected T-cells induced cytolysis of HBV-infected cells and antigen-positive HCC cells, leading to reduction of HBeAg and loss of cells expressing viral RNA [73]. IMC-I109V is currently in phase 1/2 clinical trial, involving mainly non-cirrhotic and virally suppressed HBeAg-negative CHB patients. The reported frequencies of HLA-A*02:01 are highly variable among different ethnicities. For instance, its prevalence is 11–20% in Asians and 23–60% in Caucasians [74]. Subsequently, a HLA non-restrictive approach (HLA-E) has been developed and this would allow drug target engagement in all patients [75]. It is worth noting that adverse reactions related to infusion of IMC-I109V such as hypersensitivity, anaphylaxis, and cytokine release syndrome have been reported in treatment with tebentafusp, another novel bispecific TCR-anti-CD3 directed against gp100 for patients with advanced melanoma, which might require immunosuppressive therapy (e.g., corticosteroids) for treatment [76]. Given the excellent safety profile of current antiviral treatment with NAs, it is crucial to establish the safety of T-cell therapy in CHB patients, which means achieving a safe and effective level of cytolysis as induced by the redirected T cells, which is therapeutically adequate to achieve HBsAg seroclearance but not to the level of excessive hepatocyte death, causing hepatic decompensation [77].

Therapeutic vaccines for CHB make use of different vaccination doses and frequencies to stimulate an anti-HBV immune response. Different viral components have been used, including DNA or peptide vaccines, vector or cell-based vaccines, and combinations of core, X, and polymerase antigens in addition to HBsAg. GS-4774, a yeast-based vaccine that consists of highly immunogenic recombinant HBcAg, HBsAg, and HBx epitopes, led to HBsAg decline ≥0.5 logs at week 24 in 3 out of 50 CHB patients who received it every 4 weeks, and no patient experienced a functional cure [78]. ABX-203 (HeberNasvac), a yeast-based vaccine that comprises HBsAg and HBcAg virus-like particles, was studied in phase 1–3 clinical trials. It led to HBsAg seroclearance in two out of six CHB patients who had prior PEG-IFN treatment and were given ABX-203 intranasally every 2 weeks for up to 5 years [79]. TG-1050, an adenovirus 5-based vaccine that expresses HBV polymerase and domains of core and HBsAg, was shown to induce specific IFNγ-producing T cells and led to minor reductions of HBsAg and significant reductions in HBcrAg in NA-treated CHB patients [80].

GC1102 is a recombinant monoclonal hepatitis B immunoglobulin (HBIg) with enhanced affinity to HBsAg. HBsAg seroclearance was achieved in 22.2% CHB patients whose baseline HBsAg were ≤1000 IU/mL after 7 weeks of treatment with GC1102 [81]. VIR-3434 is another recombinant HBIg that is currently being evaluated in a phase 1 clinical trial. Initial data showed that six out of eight patients achieved a mean reduction of 1.3 logs of HBsAg by day 8 [82]. It is worth noting that both trials of recombinant monoclonal antibodies recruit CHB patients with baseline HBsAg ≤ 1000 IU/mL. Similar to NAPs and STOPS, it is expected that reduction in liver and circulating viral antigen load using neutralizing antibodies will restore adaptive immunity [65]. Historically, HBIG derived from HBsAg-vaccinated subjects could lead to emergence and/or selection of immune escape HBV mutants, particularly in the ‘a’ determinant loop, that enables viral persistence despite antibody titers. Cases of fulminant hepatic failure resulting from immune escape mutants have been reported in liver allograft recipients [83,84,85,86]. This again highlights the importance of safety during drug development. The potential risk of recombinant HBIG-induced escape mutations and subsequent hepatic failure can be minimized by selecting patients with low viral load, e.g., low HBsAg titers as in the GC1102 trial, the use of next generation monoclonal antibodies that display improved broadly neutralizing potential against different HBV strains and escape mutants [87], and the concomitant use of another antiviral agent, e.g., NAs to reduce the immune selection pressure.

SB9200 (Inarigrivir) is a dual agonist of retinoic acid inducible gene-1 and nucleotide-binding oligomerization domain that consists of host PRRs that activate the innate immune pathway. SB9200-induced HBsAg decline ≥ 0.5 logs from baseline in 22% patients [88]. However, the developing company prematurely terminated the phase 2b trial after the occurrence of unexpected serious adverse events, including one patient death in the Phase 2b CATALYST trial of SB9200 in January 2020.

## 4. Combination Strategies

With the efficacy and safety data of the individual novel agents mentioned above, the approach of combining two or more novel agents to reduce viral antigen load, inhibit viral replication, and stimulate immunity is being actively explored.

The triple combination of RNAi (monthly injections for three doses of JNJ-3989) + CpAM (daily oral doses of JNJ-6379 for 85 days) + NA (daily oral doses beyond the end of CpAM dosing) led to mean 1.7 logs reduction in HBsAg on day 113 in 12 CHB patients. In addition, other viral products including HBV DNA, HBV RNA, and HBcrAg were profoundly suppressed. This combination therapy was in general well-tolerated with no serious or severe adverse events reported. Mild ALT flares were observed in five patients and were attributed to therapeutic flares [89].

A few more combination therapies are currently being evaluated in a phase 2 study that evaluates the safety and efficacy of multiple combination therapies (ClinicalTrials.gov identifier: NCT04225715). One of them is a similar example as the above with triple combination with RNAi (RG6346) + CpAM (RO7049389) + NA. Other examples include: triple combination of RNAi (RG6346) + TLR7 agonist (RO7020531) + NA, triple combination with RNAi (RG6346) + PEG-IFN + NUC, and triple combination with CpAM (RO7049389) + TLR agonist (RO7020531) + NA. The approach of sequential use of therapeutic vaccines after antigen knockdown by RNAi has been explored in mice models that showed encouraging results in suppression of viral burden and stimulation in the number of functional HBV-specific T cells and production of HBV-neutralizing antibodies [90]. Clinical studies using this approach are awaited.

Some agents will require combination with NAs, especially CpAMs, NAPs, and most immune modulatory agents, in contrast to RNAi. This is likely to be related to whether the antiviral effect of the individual agent leads to silencing of cccDNA transcriptional activity, which is the case for RNAi via post-transcriptional knockdown of HBV transcripts. Theoretically, sequential combinational therapy will result in rapid decline in HBsAg (by virus-directing agents) which is beneficial for subsequent immune stimulation therapy [91]. In transgenic mice models, GaINac-conjugated siRNA followed by therapeutic vaccine showed significantly stronger immune stimulatory effect in terms of development of polyfunctional, HBV-specific CD8^+^ T cells compared to mice given control RNAs with NA, and therapeutic vaccine [90]. Although many RNAi-based combination therapies are still tested in clinical trials in combination with NAs, it remains to be unveiled whether the viral suppressive effects of NA can be substituted by other virus-directing agents.

## 5. Conclusions

The currently preferred treatment endpoint for CHB is functional cure. Novel agents work on different steps of viral replication or the host’s immune system, in order to reduce viral burden, inhibit viral replication, and restore host immunity. The therapeutic effects of both virus-directed and host-immunity-directed agents are intercalated and are equally crucial for achieving a functional cure. Most agents currently in clinical phases of development demonstrated favorable results in suppression of viral proteins and genomic materials, with initially promising results of enhancing the functional cure of CHB. With an increasing number of novel agents entering clinical phases of development, it is expected that each agent should demonstrate favorable safety data, and long-term follow-up data in the subjects that participated in these trials will be an important consideration. Most trials aimed to achieve broad eligibility by recruiting CHB patients with variable duration of infection and viral activity. However, it is likely that specific subgroups of patients will benefit most from selected agents so that a finite duration of therapy (partial cure or functional cure if HBsAg seroclearance can be achieved) instead of lifelong NA treatment can become feasible. For all trials mentioned, no cirrhotic patients were included and this will need to be addressed when more safety data are available. Combination therapy with two or more novel agents theoretically leads to synergistic therapeutic effects. NA and/or PEG-IFN are still the backbone of these combination regimens. The best cocktail of therapies is being sought, and different regimens are likely to be needed for different patient populations with various viral factors (e.g., HBsAg levels, previous treatment exposure), and host factors (e.g., HLA allele, gender).

## Figures and Tables

**Table 1:** Definitions of therapeutic endpoints for HBV therapy.

Therapeutic Outcome	Blood				Liver	
	HBV DNA	HBsAg	Anti-HBs *	Anti-HBc	cccDNA	Integrated DNA
Partial cure	−	+	−	−/+	+	+
Functional cure	−	−	−/+	−/+	+	+
Complete cure	−	−	−/+	−/+	−	+
Sterilizing cure	−	−	−/+	−/+	−	−

* Anti-HBs and anti-HBc are not required for defining therapeutic endpoints. Anti-HBs: antibody to HBV surface antigen; cccDNA: covalently closed circular DNA; HBsAg: hepatitis B surface antigen; HBV: hepatitis B virus.

**Table 2:** Novel antiviral agents in clinical phases of development.

Main Mechanism	Remarks		Drug Names	Delivery	Phase	Clinical Trial Identifier
Inhibition of viral entry	NTCP binding		**Myrcludex B/Bulevertide**	SC	3	NCT03852719
	Cyclophilin inhibitor		CRV-431	Oral	1	NCT03596697
RNA interference	siRNA		Dicerna GAIXc-HBVS (**RG 6346**)	SC	1/2	NCT03772249
			**JNJ 3989** (ARO-HBV 1 & ARO-HBV 2)		2	NCT04129554
			**AB-729**		2	NCT04820686
			**VIR-2218** (ALN-HBV)		2	NCT03672188
	ASO		**GSK-836** (ISIS-358)-non GaINAc	SC	2	NCT04449029
			GSK-404-GaiNAc		2	NCT03020745
			RO7062931-GaiNAc		1	NCT03038113
Inhibition of capsid formation	CpAM	Class 1	**GLS-4** (Morphothiadin)/ritonavir	Oral	2	NCT04147208
		Class 2	**ABI-HB0731** (Vebicorvir)		2	NCT03780543
		Class 2	ABI-H2158		2	NCT04398134
		Class 2	**JNJ-6379**		2	NCT03361956
		Class 2	EDP-514		1	NCT04470388 NCT04008004
		Not disclosed	QL-007		1	NCT03770624 NCT03244085
		Class 2	ZM-H1505R		1	NCT04220801
		Class 2	ABI-H3733		1	NCT04271592
		Class 2	ALG-000184 (prodrug of ALG-001075)		1	NCT04536337
		Class 1	**RO7049389** (RG7907)		1	NCT02952924
Inhibition of HBsAg release	Nucleic acid polymer		**REP 2139** or REP 2165	IV	2	NCT02565719
	STOPS		ALG-010133	SC	1	NCT04485663
Interaction with host nuclear receptor	FXR agonist		**EYP001**	Oral	2	NCT04465916
Enhancement of innate/adaptive immunity	TLR agonist		RO7020531 (also known as RG-7854, TLR7)	Oral	1	NCT02956850
			Vesatolimod (TLR7, **GS-9620**)		2	NCT02166047
			Selgantolimod (TLR8, **GS-9688**)		2	NCT03491553
	T cell		ASC22 (Anti-PDL1)	SC	2	NCT04465890
			Cemiplimab (Anti-PD1)	IV	1/2	NCT04046107
			**Nivolumab** (Anti-PD1)	IV	1	ACTRN12615001133527 *
			APG-1387 (apoptosis inducer)	IV	2	NCT04568265
			IMC-I109V (soluble T-cell receptor, ImmTAV molecule)	IV	1/2	NCT03973333
	Therapeutic vaccine		HeberNasvac (**ABX-203**)	Intranasal	3	NCT02249988
			**GS-4774**	SC	2	NCT01943799
			HepTcell	IM	2	NCT04684914
			AIC649	IV	1	Not applicable
			HB-110	EP	1	NCT01641536
			VTP-300	IM	1/2	NCT04778904
			JNJ 64300535	EP	1	NCT03463369
			BRII-179 (VBI-2601)	IM	1/2	NCT04749368
			**TG-1050**	SC	1	NCT02428400
			INO-1800	EP	1	NCT02431312
	Monoclonal antibody		**GC1102**	IV	2	NCT03801798
			**VIR-3434**	SC/IV	1	NCT04423393

ASO: antisense oligonucleotide; CpAM: core protein allosteric modulator; EP: electroporation; FXR: farnesoid X receptor; HBsAg: hepatitis B surface antigen; IM: intramuscularly; IV: intravenously; NTCP: sodium taurocholate cotransporting polypeptide; SC: subcutaneously; siRNA: small interfering RNA; STOPS: S-antigen transport-inhibiting oligonucleotide polymers; TLR: toll-like receptor. * Australian New Zealand Clinical Trials Registry (with or without GS-4774). Agents in bold text represent those with clinical data available.

## Data Availability

Not applicable.
